# Women show a closer association between educational level and hypertension or diabetes mellitus than males: a secondary analysis from the Austrian HIS

**DOI:** 10.1186/1471-2458-12-392

**Published:** 2012-05-30

**Authors:** Alexandra Kautzky-Willer, Thomas Dorner, Ann Jensby, Anita Rieder

**Affiliations:** 1 Gender Medicine Unit, Department of Internal Medicine III, Division of Endocrinology & Metabolism, Medical University Vienna, Währinger Gürtel 18-20, 1090, Vienna, Austria; 2 Institute of Social Medicine, Centre for Public Health, Medical University Vienna, Rooseveltplatz 3, 1090, Vienna, Austria

## Abstract

**Background:**

Lifestyle diseases and cardiovascular complications are dramatically increasing, but little is known about the impact of educational level and health behaviour in men and women in different populations. Therefore, we aimed to investigate the association between educational level (EL) and self-reported chronic diseases and health behaviour in both sexes.

**Methods:**

Data were derived from the Austrian Health Interview Surveys 2006/2007, which includes 13 558 persons (50.9% females). The associations between EL and the risk of obesity, hypertension, diabetes, myocardial infarction, stroke and anxiety disorders or depression, nutrition, exercise, and smoking were evaluated. University education served as the reference category (EL4), the lowest educational level was required schooling only (EL0).

**Results:**

Only among women did the risk for diabetes mellitus and hypertension increase with decreasing educational level with the highest rates for EL0 OR [95% CI] adjusted for age, income, family status and lifestyle: 3.7 [1.7-8.0], and 2.5 [1.8-3.5], respectively. Only among the men, however, did the risk for stroke increase with decreasing educational level adjusted OR for EL0: 8.5 [1.7-42.7]. For anthropometric measures and lifestyle factors in both sexes the risk increased with decreasing EL.

**Conclusion:**

EL affects lifestyle, overweight and obesity in both sexes. The apparent sex-specific differences in the association between the prevalence of some chronic disease with EL call for further investigation.

## Background

Educational level is regarded as an important health determinant, with potential gender-related differences and ethnic and cultural variations. However, there is little information available regarding the impact of educational level on lifestyle, nutrition and physical activity, as well as on the presence of chronic lifestyle-diseases like overweight/obesity, hypertension, diabetes and associated cardiovascular complications in population-based studies. In addition, educational level appears related to socioeconomic status in many countries; the latter is clearly associated with obesity, which may be the underlying cause of many other diseases[1]. Therefore, for women negative associations were described between obesity and education and occupation in industrial countries, but positive associations were found with income and material resources in less developed countries [1]. Interestingly, in a recent study among Iranian adults, educational level was demonstrated to be inversely related to obesity in both sexes, but abdominal obesity, measured by waist circumference, was inversely related to educational level only among the Iranian women [2]. In addition to education, marital status, parity and age of marriage also associated with the increasing trend of obesity in the north of Iran [3]. In general, the negative association between education and obesity appears to be a more consistent finding in women [4]. Lower socioeconomic status has been shown to be associated with an unhealthier lifestyle, including increased consumption of alcohol and cigarette smoking, sedentary behaviour and less-healthful nutrition [5,6]. Health-related behaviour is associated with self-perceived health and may contribute to socioeconomic inequalities in health care [7]. Health behaviour may already be determined during childhood but was shown to relate to highest attained educational level [5,8]. In addition, gender-differences have been described in relation to health behaviour with males exhibiting more health risks and less preventive behaviour than females [9,10]. Alcohol consumption is more common in men, but in regard to smoking a dramatic increase particularly among younger females has been reported [11]. Compared to women in other European countries, younger females in Austria show the highest rate of smoking [12]. In general, health-related behaviour and life expectancy currently seem to be changing in both sexes [13].

Obesity and type 2 diabetes are rapidly increasing all over the world and are lifestyle diseases with far-reaching health complications and reduced life expectancy [14-16]. The main cause of death in both sexes is cardiovascular diseases, but diabetes is often the unrecognized but underlying cause of vascular complications. In general, males are more likely to have myocardial infarctions compared to premenopausal women, but in the presence of prediabetes or diabetes the risk for women increases dramatically and cardiovascular mortality is very high in women with impaired glucose metabolism and in younger women with cardiovascular disease [17-20]. Cardiovascular mortality has improved less in women compared to males in the last decade and various studies have shown that women are treated differently than their male counterparts when seeking related health care, although the cause of such difference is still unclear and complex [21,22]. Blood pressure in women is higher at older ages as well as low-density lipoprotein (LDL) cholesterol despite antihypertensive and statin therapy [22,23].

Nutritional habits and the degree of physical activity play an important role in the development of overweight, diabetes and many chronic diseases [24-26]. Most evaluations of the effect of meat consumption on diseases such as obesity or cardiovascular disease differentiate between red meat and poultry, and sometimes further specify whether the meat was processed [25]. Physical activity has been associated with a graded, inverse relationship with body mass index (BMI), abdominal and visceral fat, and weight gain [27-30]. Physical inactivity is an important risk factor for all-cause and cardiovascular (CV) mortality in both men and women [31,32].

Therefore, further studies exploring lifestyle factors affected or modified by education in both sexes are needed [2]. Based on this background, the aim of this study was to assess the association between lifestyle, nutrition and physical activity, and the presence of widespread chronic diseases, obesity, diabetes and hypertension and related complications with educational level in a representative sample of the population in Austria. Anxiety disorders and depression were also included because sex differences are well-known in mental health and because these common disorders also relate to obesity, diabetes and cardiovascular disease and cause high health expenditures.

## Methods

The analysis is based on the data of the Austrian Health Interview Survey (AT-HIS) 2006–07 [33], which was conducted in-person with a representative sample of Austrians over the age of 15 years. To insure inclusion of persons across the entire geographical area of Austria, the country was divided first into the 32 “care regions” (NUTS-3 regions). From each of these regions 770 persons were chosen for the sample, with the exception of the three regions in Vienna from which 990 persons were selected. In order to account for the stratification of the sample, the data were weighted by geographic region, age, and sex. From the total sample of 25 130 persons, 621 were omitted due to neutral reasons. From the remaining sample, 9035 persons (36.9%) were not included for reasons such as refusal to be interviewed, quitting in the middle of the interview, absence, etc. The response rate after excluding the neutral deficiency was 63.1%. In the following analysis, only persons between the ages of 20–79 have been included, forming a total of 13 558 cases. The questionnaire was based on the European Core Health Interview Survey (EC-HIS) [34] and was adopted by an Austrian expert panel.

The selected health behaviours analyzed here include diet rich in meat, lifestyle low in physical activity, no regular intensive exercise, overweight and obesity, and daily smoking. The survey included the means to collect anthropometric measures, as well as questions regarding health status and health behaviours. Body Mass Index (BMI) was computed as kg/m² from self-reported height and weight measurements.

### Dependent variables: health conditions, behaviours and chronic diseases

To determine the prevalence of specific diseases, respondents were asked to identify diseases that had appeared in the last 12 months and whether the disease or condition had also been diagnosed or confirmed by a doctor. Only diseases and conditions that were confirmed by a doctor were evaluated as present, in order to reduce the possibility of distortion of data due to self-diagnosis. The diseases diabetes, hypertension, myocardial infarction, stroke and anxiety disorders or depression were used in our analysis.

Overweight was defined as having a BMI of 25 kg/m² or more and obesity was defined as having a BMI of at least 30 kg/m², as proposed by the World Health Organization [35] and the National Institutes of Health [36].

Respondents were also asked about specific health behaviours such as nutritional habits, physical activity, alcohol consumption and smoking.

Smoking habits were reported in answer to the question “Do you currently smoke?” Possible answers were “yes, daily”, “yes, occasionally”, and “no”. Subjects who answered with “yes, daily” were classified as smokers. Regarding nutritional habits the subjects were asked: “Which category best describes your eating habits?” The possible answers were “Mixed diet rich in fruit and vegetables”, “mixed diet, rich in meat”, “mixed diet with little meat”, “vegetarian diet with dairy products and/or eggs”, “vegetarian with fish and/or dairy products and/or eggs”, and “vegan diet without any animal products”. For further analyses the item “mixed diet, rich in meat” was used. The physical activity habits were assessed with the question “In your leisure time do you take part in physical activity that is intense enough to make you sweat at least once a week? E.g. vigorous walking, bicycling, aerobics, etc.” The possible answers were “Yes “ and “No”. Subjects who answered “No” were classified as subjects with no regular vigorous exercise.

### Independent variables: educational level

In Austria, the minimum amount of schooling required by the state is nine years, beginning at the age of six. After the required schooling, one may continue with education or complete an apprenticeship. Options for continuing education include the *Berufsbildende mittlere Schulen* (BMS), a lower-level specialized vocational school that does not culminate in *Matura*, the required diploma for university study. The BMS education continues for an additional three years after the required amount of schooling. Another option is the *Berufsbildende höhere Schule* (BHS) which is also job-preparatory, but can also lead to the diploma required for university study.

Students can matriculate into the *Allgemeinbildende Höhere Schule* (AHS) after the fourth year of education or after the eighth year. It is considered the highest level of education before post-secondary and tertiary education and ends in the granting of the required diploma for university study.

The educational level was recorded as the highest level of education the person reported as having completed. The choices were: required schooling only (EL0); required schooling with an apprenticeship (EL1); lower level vocational school (EL2); higher-level vocational school or general secondary school, both of which can end in the diploma required for university studies (EL3); or university (EL4).

### Additional independent co-variables

As an additional co-variable, family status was assessed and dichotomized in a variable with two values: being married and living with a partner vs. being single, married but separated, divorced or widowed. Also, net household income was categorized in a variable with three values: 1 – 1200 Euro, 1201 – 2600 Euro, and 2601 Euro and more. Occupational group was assessed in four categories: white-collar worker, blue-collar worker, civil servants, and freelancers and self-employed.

### Statistical analysis

SPSS Statistics 17.0 was used for the statistical analyses. All data have been stratified for sex. Bivariate analyses were conducted by means of cross-tabs, and group differences in dichotomous dependent variables were assessed with the Pearson’s Chi²-test. In order to test the hypothesis about differences in health conditions and health behaviour by educational level, binary logistic regression models have been computed and results are reported as odds ratios with confidence intervals. In the models with health behaviour as the dependent variables, educational level together with age, occupational class, net household income, and family status were used as independent variables and odds ratios shown for educational level. In the models with chronic diseases as dependent variables, again the independent variables age, occupational class, net household income, and family status were applied, completed with the health behaviour parameters smoking, diet rich in meat, and lack of regular vigorous exercise in leisure time. Confidence intervals were calculated at the 95% level.

### Ethical considerations

The secondary analysis for this study was approved by the Ethics Committee of the Medical University Vienna (EC # 770/2011).

## Results

In total 50.9% of the participants were female. Demographic characteristics of men and women are reported in Table 1. There were more women than men in older age groups and women tended to be married more often than men. The proportion of women with required schooling only and vocational school was higher in women, but there were more men with required schooling plus apprenticeship. Women tended to have a lower net household income than men. There were more female white-collar workers, but more male blue-collar workers and civil servants. Women reported anxiety disorders or depression more often than men and a higher rate of physical inactivity but a lower rate of myocardial infarctions, overweight, daily smoking and diet rich in meat (all p < 0.001) (Table 1). Furthermore, women had a higher rate of basic education (EL0) and secondary education without diploma (EL2), but a lower rate of apprenticeship (EL1) in comparison to the male population (all p < 0.001) (Table 1). No difference was seen regarding university degree (EL4) or secondary education with diploma (EL3) between the sexes.

**Table 1:** Number of men and women and the percentage (%) of the total group studied with the respective demographic characteristics, diseases and health related conditions and behaviour

	**Age 20–59 years**			**Age 60–79 years**		
	**Men**	**Women**	***P***^**a**^	**Men**	**Women**	***P***^**a**^
**N**	**5188**	**5157**		**2465**	**1748**	
**Socio-demographic characteristics**						
**Education**						
Required schooling only	609 (11.7)	1069 (20.7)	<0.001	379 (27.1)	838 (48.0)	<0.001
Required schooling plus apprenticeship	2462 (47.5)	1516 (29.4)	<0.001	671 (45.8)	406 (23.2)	<0.001
Vocational (BMS)	526 (19.1)	907 (17.6)	<0.001	141 (9.6)	295 (16.9)	<0.001
High school (AHS/BHS)	1024 (19.7)	1008 (19.5)	0.806	127 (8.7)	135 (7.7)	0.332
University	566 (10.9)	656 (12.7)	0.004	129 (8.8)	72 (4.1)	<0.001
**Net household income**						
1 – 1200 Euro	893 (17.2)	1199 (23.3)	<0.001	388 (26.5)	754 (43.3)	<0.001
1201 – 2600 Euro	2569 (49.6)	2517 (48.8)	0.452	829 (56.7)	808 (46.4)	<0.001
2601 Euro and more	1719 (33.2)	1437 (27.9)	<0.001	245 (16.8)	180 (10.3)	<0.001
**Occupational group**						
White collar worker	1709 (39.9)	1907 (57.1)	<0.001			
Blue collar worker	1492 (34.8)	626 (18.8)	<0.001			
Civil servant	372 (8.7)	204 (6.1)	<0.001			
Free lancers and self-employees	712 (16.6)	600 (18.0)	0.118			
**Familial status**						
Being married and living with a partner (yes)	2945 (56.8)	3006 (58.3)	0.117	1193 (81.4)	963 (55.1)	<0.001
**Health conditions and health behaviour**						
Diabetes mellitus (yes)	130 (2.5)	142 (2.8)	0.431	213 (14.5)	227 (13.0)	0.207
Hypertension (yes)	669 (12.9)	615 (11.9)	0.135	658 (44.9)	830 (47.5)	0.142
Myocardial infarction	43 (0.8)	18 (0.3)	0.001	125 (8.5)	66 (3.8)	<0.001
Stroke	34 (0.7)	50 (1.0)	0.075	94 (6.4)	70 (4.0)	0.002
Anxiety disorders or depression	246 (4.7)	429 (8.3)	<0.001	103 (7.0)	208 (11.9)	<0.001
Overweight, BMI > = 25 kg/m² (yes)	2818 (54.3)	1857 (36.0)	<0.001	1035 (70.6)	1073 (61.4)	<0.001
Obesity, BMI > = 30 kg/m² (yes)	618 (12.0)	573 (11.1)	0.181	251 (17.2)	347 (20.0)	0.048
Currently daily smoking (yes)	1677 (32.3)	1288 (25.0)	<0.001	203 (13.8)	144 (8.2)	<0.001
Diet rich in meat (yes)	2273 (43.8)	821 (15.9)	<0.001	381 (26.0)	192 (11.0)	<0.001
No regular vigorous exercise in leisure time (yes)	1864 (35.9)	2231 (43.3)	<0.001	795 (54.2)	1131 (64.7)	<0.001

^a^ Pearson’s Chi²-test.

In both sexes older people tended to have to a higher proportion required schooling only and in younger subjects the proportion of subjects with high school or university degree was higher.

In women the ORs for overweight and obesity were inversely related to EL, with rates of overweight and obesity highest for EL0 (Table 2). Such trends were attenuated in males. Similar results were also seen in both sexes for the health behaviours daily smoking, diet rich in meat and sedentary lifestyle, with EL showing a stronger influence on diet in women than men (Table 3). Among men and women, significantly higher odds of daily smoking and diet rich in meat were seen at all levels of education lower than high school (Table 3). Among both men and women the odds of having a sedentary lifestyle and doing no regular vigorous exercise were significantly higher at lower levels of education than among respondents with high school or university education (Table 3).

**Table 2:** Odds ratios (OR) for the impact of educational level on chronic diseases

	**Men**			**Women**		
	**OR**	**95% CI**	***P***	**OR**	**95% CI**	***P***
**Diabetes**						
*Required schooling only*	1.37	(0.82-2.30)	0.227	3.68	(1.69-8.00)	0.001
*Required schooling plus apprenticeship*	1.31	(0.82-2.11)	0.265	2.46	(1.12-5.39)	0.025
*Vocational (BMS)*	1.15	(0.65-2.05)	0.632	2.09	(0.93-4.69)	0.073
*High school (AHS/BHS)*	1.85	(1.10-3.12)	0.020	1.85	(0.80-4.31)	0.153
**Hypertension**						
*Required schooling only*	1.42	(1.06-1.90)	0.019	2.54	(1.84-3.51)	<0.001
*Required schooling plus apprenticeship*	1.59	(1.23-2.06)	<0.001	1.69	(1.23-2.34)	0.001
*Vocational (BMS)*	1.09	(0.80-1.51)	0.581	1.47	(1.06-2.06)	0.022
*High school (AHS/BHS)*	1.51	(1.13-1.41)	<0.001	1.27	(0.89-1.81)	0.194
**Myocardial Infarction**						
*Required schooling only*	2.08	(0.94-4.63)	0.071	4.42	(0.50-39.00)	0.180
*Required schooling plus apprenticeship*	2.01	(0.94-4.31)	0.074	3.90	(0.44-34.81)	0.223
*Vocational (BMS)*	1.58	(0.64-3.92)	0.321	3.52	(0.38-32.61)	0.268
*High school (AHS/BHS)*	1.80	(0.76-4.28)	0.184	2.11	(0.20-22.24)	0.535
**Stroke**						
*Required schooling only*	8.45	(1.67-42.66)	0.010	1.06	(0.47-2.41)	0.887
*Required schooling plus apprenticeship*	7.77	(1.57-38.52)	0.012	0.75	(0.32-1.74)	0.501
*Vocational (BMS)*	7.70	(1.59-38.53)	0.017	0.54	(0.21-1.36)	0.192
*High school (AHS/BHS)*	7.03	(1.32-37.56)	0.023	0.74	(0.28-1.92)	0.737
**Anxiety disorders or depression**						
*Required schooling only*	0.69	(0.44-1.08)	0.107	1.18	(0.81-1.72)	0.404
*Required schooling plus apprenticeship*	0.67	(0.44-0.98)	0.042	1.07	(0.74-1.56)	0.713
*Vocational (BMS)*	0.83	(0.51-1.37)	0.467	0.99	(0.67-1.47)	0.964
*High school (AHS/BHS)*	0.62	(0.39-0.99)	0.047	1.07	(0.72-1.59)	0.754
**Overweight**						
*Required schooling only*	1.82	(1.45-2.28)	<0.001	2.79	(2.24-3.47)	<0.001
*Required schooling plus apprenticeship*	1.77	(1.47-2.13)	<0.001	1.56	(1.27-1.93)	<0.001
*Vocational (BMS)*	1.42	(1.13-1.78)	0.002	1.62	(1.31-2.02)	<0.001
*High school (AHS/BHS)*	1.31	(1.07-1.61)	0.009	1.10	(0.88-1.37)	0.426
**Obesity**						
*Required schooling only*	1.82	(1.27-2.61)	<0.001	2.83	(1.96-4.08)	<0.001
*Required schooling plus apprenticeship*	2.17	(1.57-2.98)	<0.001	1.65	(1.15-2.38)	0.007
*Vocational (BMS)*	1.26	(0.85-1.87)	0.244	1.81	(1.24-2.63)	0.002
*High school (AHS/BHS)*	1.36	(0.95-1.96)	0.095	1.20	(0.81-1.79)	0.371

Results of a binary logistic regression analysis, adjusted for age, occupational group, net household income, family status, smoking status, eating regularly a diet rich in meat, and lack of regular vigorous exercise in leisure time with the reference category of university education.

**Table 3:** Odds ratios (OR) for the impact of educational level on health behaviours

	**Men**			**Women**		
	**OR**	**95% CI**	***P***	**OR**	**95% CI**	***P***
**Currently daily smoking**						
*Required schooling only*	3.02	(2.30-3.53)	<0.001	2.80	(2.12-3.70)	<0.001
*Required schooling plus apprenticeship*	2.82	(2.22-3.58)	<0.001	2.86	(2.21-3.71)	<0.001
*Vocational (BMS)*	2.20	(1.66-2.91)	<0.001	2.23	(1.70-2.93)	<0.001
*High school (AHS/BHS)*	1.30	(1.00-1.69)	0.052	1.42	(1.08-1.87)	0.012
**Diet rich in meat**						
*Required schooling only*	1.61	(1.29-2.02)	<0.001	2.60	(1.90-3.56)	<0.001
*Required schooling plus apprenticeship*	1.55	(1.29-1.87)	<0.001	2.14	(1.58-2.89)	<0.001
*Vocational (BMS)*	1.40	(1.12-1.76)	0.004	1.67	(1.22-2.30)	0.002
*High school (AHS/BHS)*	1.10	(0.90-1.36)	0.349	1.30	(0.94-1.80)	0.117
**No regular vigorous exercise in leisure time**						
*Required schooling only*	2.59	(2.07-3.24)	<0.001	2.79	(2.27-3.42)	<0.001
*Required schooling plus apprenticeship*	1.46	(1.20-1.77)	<0.001	1.52	(1.25-1.85)	<0.001
*Vocational (BMS)*	1.58	(1.25-1.99)	<0.001	1.39	(1.14-1.71)	0.001
*High school (AHS/BHS)*	1.01	(0.82-1.26)	0.898	1.20	(0.97-1.47)	0.089

Results of a binary logistic regression analysis, adjusted for age, occupational group, net household income and family status, with the reference category of university education.

However, in women only we also found a strong relationship between education and diabetes mellitus, and hypertension (Table 2). The OR for diabetes was significantly higher for vocational school and required schooling with or without apprenticeship in women (Table 2).

Interestingly, in men only a weak association was found between high school (EL3) or required schooling plus apprenticeship (EL1) and hypertension as well as high school (EL3) and diabetes when compared to men with a university degree (Table 2). However, stroke was strongly related to education in males only, with low education (EL0 and EL1) bearing a 7-8-fold increased probability (Table 2). Also of note, no association was found between educational level and myocardial infarctions for either of the sexes (Table 2).

## Discussion

In both men and women in this study lower educational levels were associated with unfavourable health behaviours, overweight and higher cardiovascular risk. In both men and women the odds of overweight and obesity decreased with increasing educational level. The same was true for the odds of daily smoking, eating a diet rich in meat and doing no regular vigorous exercise. However, the association between education and the chronic diseases diabetes and hypertension may be of greater magnitude in women. Among the women of the sample, the odds of suffering from diabetes or from hypertension decreased gradually with increasing educational level. In the men, however, there was no clear association between educational level and the risk of diabetes or hypertension. Depression was increased only in women with required schooling and showed no relationship with education in men. Overall, among men, variables other than health behaviours less clearly showed an increase in probability in the lower educational levels.

Overweight and obesity are dramatically increasing in all European countries and are associated with many health complications [15,26,37,38]. In comparison to other countries Austria shows a less prominent increase of overweight and obesity in both sexes [14]. In the present survey the difference between sexes was similar to data obtained in 2004 [39] with comparable rates of obese men and women and with 25 to 30% more overweight men than women.

A significant negative association between education and general obesity was also shown in other population-based studies in both sexes with a dose–response relationship from illiterate and primary education to high educational levels [2]. Furthermore, prevalence rates of both overweight and obesity were highest in women with low incomes, though such a relationship was not characteristic of the male population in Europe [14]. However, the inverse relation between social class and obesity in women in wealthy countries contrasts with the findings in low-income countries [40,41]. Overweight increased with increases in education levels in poor rural populations in India, where overweight is also seen as sign of wealth and health [41]. Overall, our findings are in agreement with previous reports confirming a stronger association between obesity and low socioeconomic status as proxy indicator of education or education levels in women in Europe.

As with obesity, diabetes is a growing problem worldwide with significant social and economic impact. In Austria at least 5% of the population is estimated to be affected [42]. In the U.S., in counties with a high risk of diabetes, thirty percent of excess risk was ascribed to obesity and sedentary lifestyle while 37% was attributed to non-modifiable risk factors such as age, gender, ethnicity and education [43]. The Multi-Ethnic Study of Atherosclerosis revealed inverse socioeconomic gradients in hypertension, diabetes, overweight and smoking in particular in white and black women and in white men, although in the latter the associations were weaker, but stronger for education than income [44]. In non-white men higher socioeconomic status was related to higher BMI, further supporting ethnic and gender differences in social patterning. A survey from Argentina reported that among women higher education was associated with better risk factor profiles including diabetes and hypertension and lower BMI in all areas but more strongly in urban areas [45]. Among men in less urban areas no association or even an adverse association was found between education and these risk factors. In Northern Italy, in men a low level of education (defined as falling into the lower of two educational categories) was related to higher BMI, prevalence of diabetes and smoking [46]. Less-educated women showed higher blood pressure and BMI and in both sexes of the low educational class a twofold increased incidence of stroke and cardiovascular disease (CVD) was observed at follow-up. However, in men CVD incidence alone was not related to education and in women higher ischemic stroke rates were observed in the more-educated group.

Of note, in our analysis stroke only was found to be related to low education in men while no association was evident in women. In another study, more physical activity at work and during leisure time was found to be associated with a lower incidence of stroke [47]. Lower socioeconomic status and less education appear to be associated with less knowledge of risk factors of stroke in both sexes [48] but in general women seem to have better knowledge of warning signs than men. Also, men more frequently mentioned stress, physical activity and smoking as risk factors of stroke, while women more often reported diabetes and hypertension, which may also be attributed to the more frequent medical visits of women.

Smoking is a leading cause of morbidity and mortality in many industrialized countries, including Austria. Various studies reported a trend towards equalisation of smoking behaviour between the sexes, showing an increase in women but a decrease in men [11]. The higher rate of smokers in the low education group in our evaluation is in line with other reports.

In a large prospective study of elderly people, red and processed meat intakes were associated with modest increases in total mortality, cancer mortality, and cardiovascular disease mortality [49]. Data on education level and red meat intakes are scarce. However the relationship between low education level and high intake of red meat fits the overall finding of unhealthier lifestyles in people with lower socioeconomic status and educational levels.

In Austria all residents have health insurance coverage; the access to health care should be comparable among groups. In addition, education is free of charge, including university studies. Quality of life and standards of health care are high in Austria. The gender gap in salaries is, however, rather high, approaching 20 to 30%, although rates of tertiary education are nowadays comparable between women and men. This trend of equalisation in tertiary education between sexes has mainly been achieved in the last decade and higher-educated women can therefore be expected to be younger than males at present, a fact that might influence the prevalence rates of diseases like diabetes or cardiovascular disease in highly educated men and women. Nevertheless, in our analysis the impact of age is negligible because all data analyses have been corrected for differences in age.

Low educational level, in particular required schooling only, is usually associated with low income in both sexes and thus lower socio-economic status may be expected in men and women with required schooling only. In Austria, as in most countries, more women than men are living below the poverty line and it has also been shown that expenditures on health care and out-of-pocket healthcare payments constitute a much greater proportion of the household income among poor people compared to the better-earning population [50]. It was shown that in particular for older persons, the lower the education the greater the burden for medical services and the lower the awareness of how to lead a healthy lifestyle, and the lower the adherence to medication and the utilisation of preventive measures. Furthermore, in women the burden for medical services was even greater than in men including income-independent disadvantages such as sex-specific illnesses. In general, in Austria as well as in other European countries women participate more often in screening programs, are more interested in health prevention and visit their general practitioners more often. These attitudes may also relate to a higher rate of diagnosis of depression and anxiety disorders. Along with biological factors including sexual hormones, in particular oestradiol, psychosocial factors, culture and education may be responsible for the sexual-dimorph patterns of these mental disorders. A recent meta-analysis confirmed that less education is generally an important risk factor for late-life depression [51]. In addition, poor self-reported health status appeared to be more strongly associated with depression than the presence of chronic disease [52]. In the Korean Longitudinal Study of Aging, which used a nationally representative sample of community-residing adults aged 45 and older, the authors found that cognitive ability, economic resources, social status, social network, and health behaviour explained all of the education gradients associated with depression [53]. Epidemiological studies indicate that the lifetime risk of depression is twice as high in women as in men, and socioeconomic status, stressful life events and biological factors contributed to higher female vulnerability and predominance of the disease [54]. Also in our study the rate of depression and anxiety disorders was twice as high in females as males and related to poor education (EL0) in women, but not in men. Therefore, our results further support the hypothesis that education – potentially reflecting cognitive ability and socioeconomic status – more strongly influences mental health in female.

This study has several limitations. Based on the cross sectional design of the study no causal explanations are possible. In addition the validity of self-reported data regarding information on dietary habits, the degree of physical activity, and smoking, as well as self-reported data about chronic diseases may have a limited reliability.

Overall, lifestyle modification leading to healthier behaviour and better health awareness and greater participation in screening and prevention strategies should be further encouraged in the most vulnerable groups: people with low education, particularly females with low education. The next decades will show if the increasing number of persons with high education currently apparent in European countries in both genders will be associated with improved health literacy and health status of both men and women.

## Conclusions

Based on these data, education affects lifestyle, smoking and overweight in both genders; low education had stronger impact on obesity and diabetes in women, but on stroke in males. Hypertension was influenced by education in females only. Overall, health status in females seems to be more closely related to educational level. Although educational level is associated with cardiometabolic risk in both sexes, these relationships seem to be differently regulated in men and women.

## Competing interests

The authors declare that they have no competing interests.

## Authors' contributions

AK-W wrote the manuscript, TD and AJ made substantial contribution to acquisition of data and analysis and interpretation of data and AR was involved in drafting the manuscript and revising it critically for important intellectual content. All authors read and approved the final manuscript.

## Pre-publication history

The pre-publication history for this paper can be accessed here:

http://www.biomedcentral.com/1471-2458/12/392/prepub
